# Integrated analyses of transcriptome and metabolome provides new insights into the primary and secondary metabolism in response to nitrogen deficiency and soil compaction stress in peanut roots

**DOI:** 10.3389/fpls.2022.948742

**Published:** 2022-09-28

**Authors:** Liyu Yang, Qi Wu, Haiyan Liang, Liang Yin, Pu Shen

**Affiliations:** Chinese National Peanut Engineering Research Center, Shandong Peanut Research Institute, Shandong Academy of Agricultural Sciences, Qingdao, China

**Keywords:** peanut root, soil compaction stress, nitrogen deficiency, metabolome, transcriptome

## Abstract

Peanut (*Arachis hypogaea* L.) is an important oil crop globally because of its high edible and economic value. However, its yield and quality are often restricted by certain soil factors, especially nitrogen (N) deficiency, and soil compaction. To explore the molecular mechanisms and metabolic basis behind the peanut response to N deficiency and soil compaction stresses, transcriptome and metabolome analyses of peanut root were carried out. The results showed that N deficiency and soil compaction stresses clearly impaired the growth and development of peanut's aboveground and underground parts, as well as its root nodulation. A total of 18645 differentially expressed genes (DEGs) and 875 known differentially accumulated metabolites (DAMs) were identified in peanut root under differing soil compaction and N conditions. The transcriptome analysis revealed that DEGs related to N deficiency were mainly enriched in “amino acid metabolism”, “starch and sucrose metabolism”, and “TCA cycle” pathways, while DEGs related to soil compaction were mainly enriched in “oxidoreductase activity”, “lipids metabolism”, and “isoflavonoid biosynthesis” pathways. The metabolome analysis also showed significant differences in the accumulation of metabolisms in these pathways under different stress conditions. Then the involvement of genes and metabolites in pathways of “amino acid metabolism”, “TCA cycle”, “lipids metabolism”, and “isoflavonoid biosynthesis” under different soil compaction and N deficiency stresses were well discussed. This integrated transcriptome and metabolome analysis study enhances our mechanistic knowledge of how peanut plants respond to N deficiency and soil compaction stresses. Moreover, it provides new leads to further investigate candidate functional genes and metabolic pathways for use in improving the adaptability of peanut to abiotic stress and accelerating its breeding process of new stress-resistant varieties.

## Introduction

Peanut (*Arachis hypogaea* L.) is an important oil and economic crop in China, whose total production and exports are ranked first worldwide, making a pivotal contribution to global oilseed production and trade (Yu, 2008; Bertioli et al., 2016). In recent years, with more agricultural mechanization and intensive management of farmland, as well as other reasons, there is a trend of deteriorating soil properties of major farmlands (Tracy et al., 2011; Bottinelli et al., 2014). In particular, soil compaction has increased, and the loss of nutrients has significantly accelerated, which has negatively impacted the growth of peanut's root system and its nutrient absorption, thus influencing the yield and quality of peanut crops. The detrimental effects of soil compaction and nitrogen (N) deficiency stresses on the growth and yield of peanut are increasingly serious and solutions are in urgent need.

Soil compaction causes significant changes in soil properties, with increased mechanical resistance, poor aeration, reduced microbial activity, lower water content, and reduced availability of soil nutrients (Abbott and Murphy, 2007). Among them, the increased mechanical resistance of the soil caused by soil compacting leads to the obstruction of root development, which is the main factor of soil compacting affecting plant growth (Tracy et al., 2011; Shen et al., 2016). As a prominent abiotic stress in modern agricultural production, soil compaction reduces agricultural crop yield globally by disrupting a variety of metabolic and physiological processes, such as ionic homeostasis, ROS (reactive oxygen species) balance, and membrane system stability (Botta et al., 2010; Arvidsson et al., 2014). Soil compaction stress can induce the adaptive growth of crops, while the genetic control of root development may be closely associated with this process. In rice, *DEEPER ROOTING 1* (*DRO1*) was found to regulate the gravitropic response of roots' growing angle; the rice genotype with the functional *DRO1* allele was established better in compacted soils than the genotype without the allele (Ramalingam et al., 2017). By comparing the root phenotypes of wild-type rice and ethylene-insensitive mutants (*osein2* and *oseil1*) in compacted soil, Pandey et al. (2021) demonstrated that the roots of *osein2* and *oseil1* penetrated the compacted soil more effectively than those of the wild-type; hence, plant roots can adjust to external soil compaction by continuously releasing ethylene outward, while sensing its accumulation of ethylene around the root system, thereby modifying their root extension direction accordingly. Although recent progress has been made, the glaring gap yet to be filled in understanding the molecular mechanisms of belowground crops is how they deal with soil compaction stress. Furthermore, many metabolic processes in plant roots are greatly affected by soil compaction. Root respiration is a vital metabolic process that provides energy for root growth. The tricarboxylic acid (TCA) cycle and the cytochrome pathway (CP) are the core respiratory pathways in plant cells. In soybean, the significant decreases of isocitrate dehydrogenase in the TCA pathway and cytochrome c oxidase in the CP pathway occurred under a high-level soil compaction condition, thus inhibiting plant root growth (Wang et al., 2019). Peanut is the quintessential belowground crop, and its development process and yield could be significantly affected by soil compaction stress. For example, in work by Shen et al. (2016), root biomass and peanut yields, respectively increased by 7.5 and 4.6% when the soil bulk density was reduced by 0.1 g cm^−3^. The impact of soil compaction stress is well studied for other crops, such as cucumber, tomato, and maize (Tracy et al., 2013; Wang et al., 2013), but is far less known for peanut. In particular, research on the molecular and metabolic mechanisms underpinning how peanut plants respond to soil compaction stress is still virtually unknown.

Soil humidity and fertilizer dosage are two key factors that affect a farm crop's yield and quality. In earlier research, crops grown on highly compacted soil have been found to have a lower uptake of nutrients (Arvidsson, 1999). This could be due to both effects of compaction on roots and impacts on soil physicochemical qualities that restrict the availability of nutrients. Abundant N in soil is essential for the growth and development of plants (Lea and Morot-Gaudry, 2001; Ye et al., in press). Greater soil compaction was found to significantly reduce crop biomass, especially under a high N-application condition (Douglas and Crawford, 1993). Therefore, N deficiency stress is almost universal to plants growing in compacted soil. Different crops have evolved complicated physiological and biochemical adaptations to cope with insufficient N (Chun et al., 2005; Garnett et al., 2009; Zhang et al., 2021). For instance, in *Brassica napus*, changes in the expression levels of cell wall-related genes contributed to longer and thinner main and lateral roots that had been caused by N deficiency (Qin et al., 2018). In rice, when soil N was at a low concentration, both NADP and citrate accumulated and the TCA cycle ramped up to produce more energy and α-ketoglutarate, which improved both N transport and assimilation (Xin et al., 2019). Peanut productivity in the majority of growing regions strongly depends on the local availability of an adequate soil N supply. Yet N deficiency was recently reported to negatively influence fundamental peanut growth traits, resulting in shorter root hairs, yellow leaves, dwarf plants, and poor overall yield and quality (Li et al., 2021). At present, most studies of N deficiency have focused on the cultivation and production of peanut, leaving relatively few investigating this plant's response to N deficiency across soil compaction conditions at molecular and metabolic levels.

In this present study, we report on the comprehensive analyses of potential mechanisms in peanut root's response to N deficiency and soil compaction stress based on the transcriptomic and metabolomic results. This study elucidated the physiological and molecular responses of peanut to varied soil compaction and soil N conditions. Critical genes and essential metabolic pathways were identified in the complex regulatory mechanisms associated with these two stresses. These findings provide valuable knowledge to facilitate peanut breeding for improving N deficiency and soil compaction resistance.

## Materials and methods

### Plant materials, growth conditions, and sequencing sample preparation

Plants of the common peanut cultivar “Huayu22” were grown in the greenhouse of the Shandong Peanut Research Institute, China. The mean (± SE) growth temperature was 25 ± 3°C under a 16-h light (15000 lx)/8-h dark cycle and the relative humidity was 70 ± 5%. The basic soil total N content and available N (alkali-hydrolyzable N) content were 0.35 g/kg and 45.2 mg/kg, respectively, which were considered a low N level according to the National Soil Nutrient Classification Standard of China. In this study, the N fertilizer of 112.5 kg ha^−1^ was applied according to the local fertilization suggestion. The two soil compaction levels were set up according to the local peanut field and the previous study results, while a bulk density of 1.2 g cm^−3^ was considered the rational soil compaction condition, and a bulk density of 1.6 g cm^−3^ was considered as the soil compaction stress condition. Therefore, two soil compaction levels (T1: 1.2 g cm^−3^; T2: 1.6 g cm^−3^) and two N application conditions (N0: 0 kg ha^−1^, N1: 112.5 kg ha^−1^) were set up. Next, the peanut seeds were sown and grown under four treatment combinations: low soil compactness and nitrogen deficiency (T1N0), high soil compactness and nitrogen deficiency (T2N0), low soil compactness and appropriate nitrogen (T1N1), and high soil compactness and appropriate nitrogen (T2N1). Samples of the peanuts under these four growing conditions were collected at the seedling stage (35 days after seedling emergence) of peanut development. The peanut plants grown under four experimental conditions (T1N0, T2N0, T1N1, T2N1) were collected at the same time during the seedling period (Supplementary Figure S1). Under the same experimental condition, root samples collected from three randomly selected peanut plants were mixed uniformly as one replicate. Each experimental treatment had three replicates. After collecting, root samples were immediately frozen in liquid nitrogen until total RNA and metabolite extraction.

### Phenotypic measurements

Roots were collected for analysis from peanut plants cultivated under 4 different experimental conditions (T1N0, T2N0, T1N1, T2N1) for phenotypic analysis. Peanut root samples were thoroughly washed and spread within a thin layer (1–2 mm) of water on a transparent tray. Then the roots were scanned (modified Optical Scanner STD 4800, Epson, Japan), and the ensuing image was analyzed for three morphological characteristics: root length, root surface area, and root volume, using WinRHIZO Regular 2009 software (Regent Instruments Inc., Canada). Next, the root samples were oven-dried to the constant weight at < 70°C and weighed with a three-digit balance. SPAD value is a measure of the chlorophyll and N content of leaves, which was determined with a SPAD chlorophyll meter (SYS-SPAD-502Plus, Japan).

Root tissues with nodule attachments were dehydrated using an alcohol gradient, and then tissues were transparent using xylene and paraffin. The transparent tissue was embedded in paraffin wax (60°C) and then sliced through a microtome (Leica, RM 2016, Germany). Each sliced tissue sample was de-waxed by baking and immersed in Harris hematoxylin stain for 3 min, washed with water, and then fractionated with 1% hydrochloric acid alcohol for 15 min. The sections were dehydrated using anhydrous ethanol, transparent in xylene, air-dried, and sealed with neutral gum. Sections were colored with Safranine Malachite Green contrast methods and then visualized and photographed under a microscope (Olympus, BX53, Japan).

### RNA extraction and sequencing

Total RNA was extracted from each sample by using Trizol (Invitrogen, Burlington, ON, Canada), according to the manufacturer's protocol, and divided into two parts. After purification, RNA was fragmented at a certain temperature and ion environment. The first-strand cDNA was then synthesized using random primers and reverse transcriptase of the TruSeq Stranded kit, and the double-strand cDNA was subsequently synthesized by DNA polymerase I and RNaseH. The ensuing double-stranded cDNA products were then joined with “A” bases and adaptors. The ligated products were amplified and the final cDNA library was obtained after purification. Here, libraries of three biological repetitions were constructed for each sample. Finally, all the constructed libraries were independently sequenced by using Illumina HiSeq4500 (San Diego, CA, USA) at Majorbio, Shanghai, China.

### Identification and quantitation of unigenes

After sequencing, clean reads of each library were obtained by filtering out any reads of low quality, with contaminated joints, or having a high N content. The clean reads were then mapped onto the reference genome of cultivated peanut (*Arachis hypogaea* L.) (http://peanutgr.fafu.edu.cn/Download.php) (Zhuang et al., 2019). Clean reads were then assembled into transcripts by using StringTie (v2.1.2) and cufflinks (v2.2.1) software. The unit of measurement was TPM (Transcripts Per Million reads).

### Functional annotation and biological pathway analysis of unigenes

To understand the functions of unigenes, unigenes were subjected to a GO annotation analysis performed by Blast2GO (v2.5) software. Next, the unigenes were classified accordingly into different categories by using WEGO2.0 (Web Gene Ontology Annotation Plotting) website. KEGG (Kyoto Encyclopedia of Genes and Genomes) is a public database that provides information about the biological pathways that genes are involved in. The pathways of unigenes were annotated by submitting their sequences to the KEGG database via BLASTx (*E*-value < 0.00001). Then DESeq2 software (v1.24.0) was used to analyze the differentially expressed genes (DEGs), designated as those with a threshold |log2 fold-change|≧1 and adjusted *p*-value < 0.05. To distinguish the most relevant functions and biological pathways of unigenes under the four treatment combinations of N deficiency and soil compaction, GO and KEGG pathway enrichment analyses were implemented for DEGs by the Fisher test. The calculated *p*-value of a given GO term undergoes a Bonferroni correction while the corresponding *p*-value of the KEGG pathway undergoes a Benjamini and Hochberg correction. If a GO term or KEGG pathway had a corrected *p*-value < 0.05, it was considered significantly enriched.

### Metabolites' extraction and analysis

Fifty milligrams of each freeze-dried ground sample were homogenized in a 400-mL extraction buffer (methanol: water, 4: 1) and mixed well. Each mixture was treated with the high-throughput tissue crusher Wonbio-96c (Shanghai Wanbo Biotechnology Co., Ltd., China) at 50 Hz for 6 min at −20°C, followed by vortexing for 30 sec and ultrasonication at 40 kHz for 30 min at 5°C. To precipitate the proteins, each mixture was placed at – 20 °C for 30 min. After centrifuging the mixture at 12000 rpm for 15 min, the ensuing supernatant was subjected to LC-MS/MS analysis. Extractions were made using six biological replicates of each sample.

To ensure the reproducibility of the analysis, pooled quality control samples (QC) were generated by combining equal quantities of all samples. These QC samples were analyzed similarly to the empirical samples, in order to monitor the stability of a sample under testing. The metabolites were separated by chromatography carried out by an ExionLC^TM^AD system (AB Sciex, USA) with an ACQUITY UPLC BEH C18 column (100 × 2.1 mm i.d., 1.7 μm; Waters, Milford, USA). Solvent A consisted of water with formic acid (0.1%); solvent B consisted of 0.1% formic acid in acetonitrile: isopropanol solution (1: 1, v/v). Each 20-μL sample was injected with the flow rate set to 0.4 mL min^−1^. Samples were separated by different solvent gradients. The column temperature was maintained at 40°C. During this analysis, all samples were stored at 4°C. The sample mass spectrometry signal was acquired in the positive and negative ion scanning mode. The operating conditions were as follows: source temperature set to 500°C; curtain gas was 30 psi; ion source GS1 and GS2 were 50 psi; the negative mode of ion-spray voltage floating was – 4000 V and the positive mode of ion-spray voltage floating was 5000 V; a declustering potential of 80 V; rolling collision energy of 20–60 V rolling for MS/MS. Metabolite data were acquired in the “Data Dependent Acquisition' mode. The detection was carried out over a mass range of 50–1000 m/z.

After the UPLC-TOF/MS analysis, the obtained raw data was loaded into the Progenesis QI v2.3 software (Nonlinear Dynamics, Waters, USA) for peak detection and alignment. The preprocessing results generated a data matrix that consisted of the retention time (RT), mass-to-charge ratio (m/z) values, and peak intensity. To distinguish the accumulation of metabolites in the different treatment samples, their raw data were processed after applying missing value filtering (>20%), missing value recording (the padding method was minimal), data normalization (normalized by summation), and data transformation (log10 transformation), via the online Majorbio Cloud Platform (www.majorbio.com) (Ren et al., 2022). Accurate mass was used to identify the mass spectra of metabolic features. Metabolite information was acquired by matching the MS/MS fragments' spectra and isotope ratio differences to the Human Metabolome Database (HMDB) (http://www.hmdb.ca/) and Metlin database (https://metlin.scripps.edu/) (Wishart et al., 2013; Zhu et al., 2013). Then, correlation analysis and principal component analysis (PCA) of the metabolites in different treatment samples were performed after their data normalization. Differential accumulated metabolites (DAMs) were defined as those metabolites having a *p*-value < 0.05 (obtained from a two-tailed Student's *t*-test) and VIP > 1 (obtained from the OPLS-DA model) (Saccenti et al., 2014). For the metabolic pathway classification and enrichment analyses of DAMs in the different comparisons, the KEGG database was used; a pathway with a *p*-value < 0.05 was considered as an enriched metabolic pathway.

### Quantitative real-time PCR

To analyze the expression of genes, twelve genes were randomly selected for validation. Total RNA was extracted from peanuts roots using Trizol (Invitrogen, Burlington, CA). After adding RNase-free DNase I to eliminate DNA contamination, total RNA was reverse-transcribed to the cDNA by random hexamer primers. The Quantitative Real-time PCR (qRT-PCR) was run on the ABI Step One Plus Real-Time PCR System (Applied Biosystems, USA) with the TB Green™ Premix Ex Taq™ II (Tli RNase H Plus) (TaKaRa, Tokyo, Japan). AhACT11 gene was chosen as the internal control to standardize the qRT-PCR results (Chi et al., 2012). The comparative Ct method (2^−ΔΔCT^) was used to calculate the relative gene expression level (Livak and Schmittgen, 2001). For all reactions, triplicate biological repetitions of each individual were performed. The results are presented as the mean ± SD of three repetitions after normalizing the data. The primers used in the reverse-transcription experiment and all qRT-PCR experiments are listed in Supplementary Table S1.

## Results

### Phenotypic changes of peanut root under low N and soil compaction stresses

**Figure 1:**
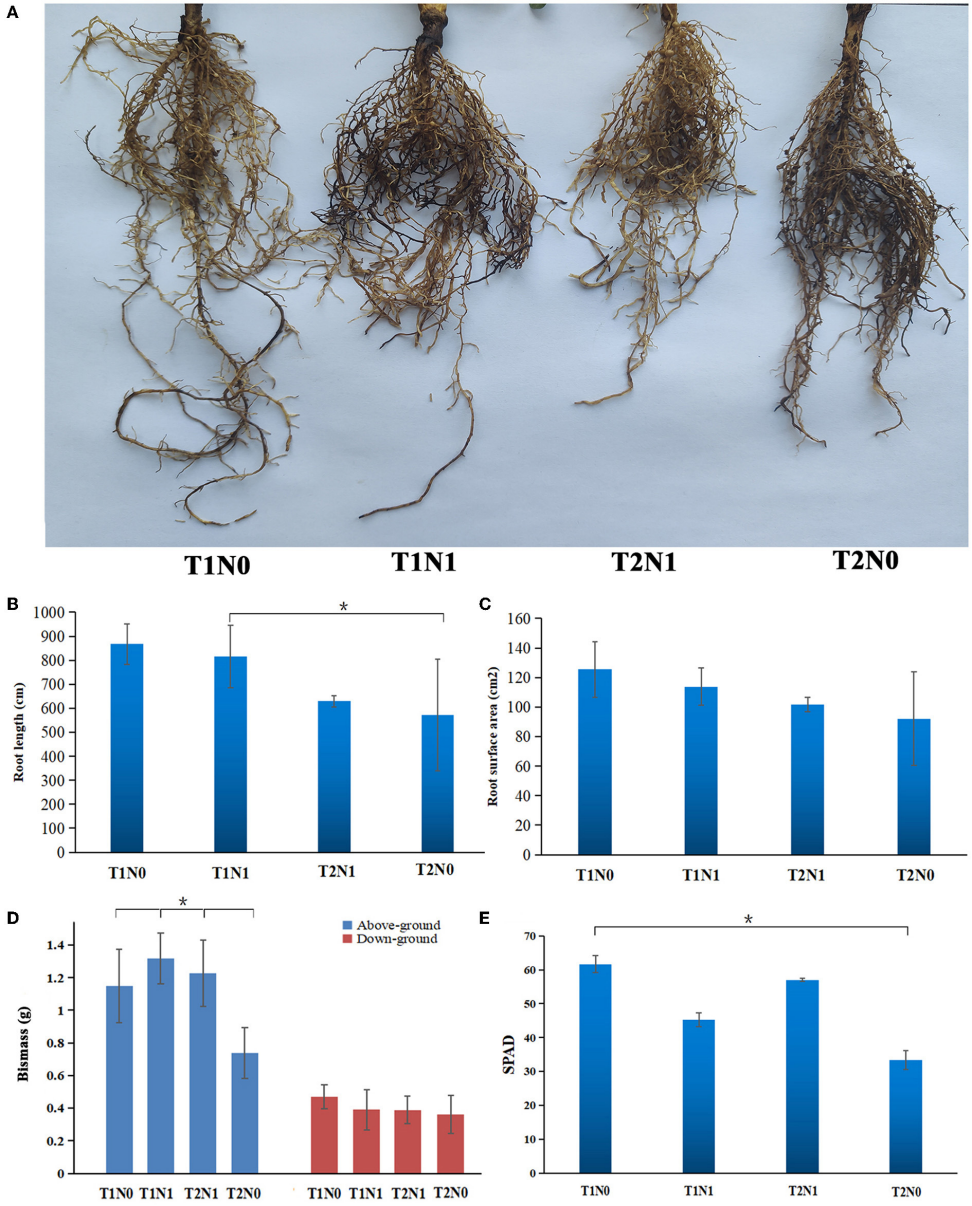
Phenotypic analysis of peanut plants grown in four different experimental conditions (treatment combinations: T1N0, T1N1, T2N0, T2N1). **(A)** Root morphology of peanut plants grew under four experimental conditions. **(B)** Length of peanut root. **(C)** Surface area of peanut roots. **(D)** Aboveground and underground biomass of peanut plants. **(E)** SPAD value of peanut plants grew under four experimental conditions. Error bars represent the SD (*n* = 3). Statistical significance was determined via an LSD test at *p < 0.05. T1N0 (Low soil compactness and nitrogen deficiency), T2N0 (High soil compactness and nitrogen deficiency), T1N1 (Low soil compactness and appropriate nitrogen) and T2N1 (High soil compactness and appropriate nitrogen).

The mechanical resistance encountered by the root system and by the soil N content directly affected the root morphology and distribution of peanut. As evinced by Figures 1A–C, in uncompacted soil, peanut plants that grew under the N deficiency condition had longer and thicker roots and a larger root volume. Under the same N nutritional condition, the higher the soil compaction, the smaller root length and surface area of peanut. Soil compaction and N deficiency stresses constrained the growth and development of the root system considerably, which influenced the formation of aboveground parts of peanut. As Figure 1D shows, under the same N nutritional condition, the aboveground biomass of peanut decreased in the high-level compacted soil. Compacted soil not only affected the aboveground biomass of peanut but also its chlorophyll (SPAD value); as shown in Figure 1E, with a lower soil N content, the SPAD value decreased. Under the same soil N content, the higher the soil compaction, the lower the SPAD value was.

**Figure 2:**
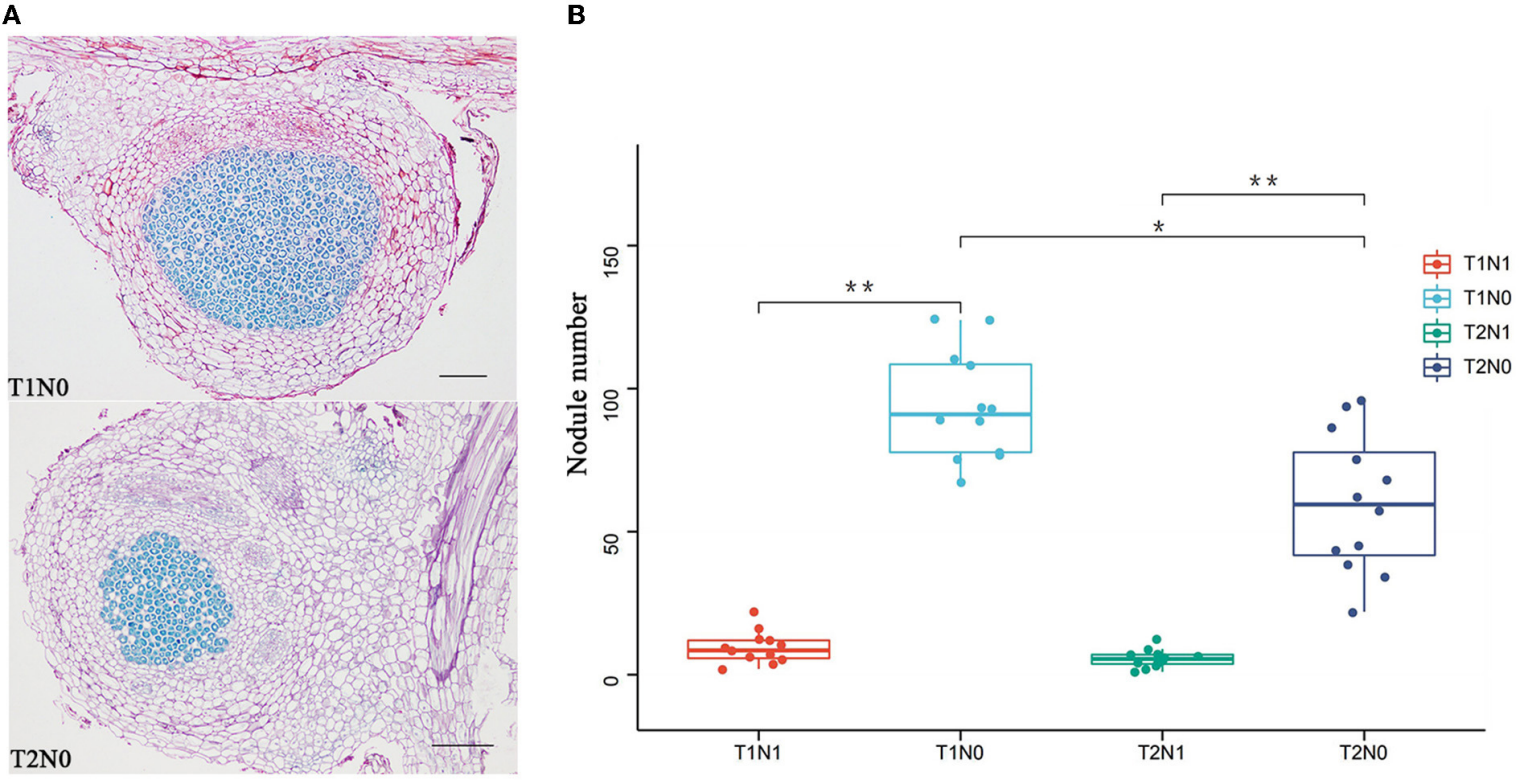
Phenotypic analysis of nodules of peanut plants grown in four different experimental conditions. **(A)** Section morphology of nodules of peanut plant grown in uncompacted (T1N0) and compacted (T2N0) soil. The red part of the section is the cortical area of the nodule, and the blue part is the bacteroid area. **(B)** Number of nodules in peanut plants. Statistical significance was determined via Student's *t*-test; *p < 0.05, ** *p* < 0.01. T1N0 (Low soil compactness and nitrogen deficiency), T2N0 (High soil compactness and nitrogen deficiency), T1N1 (Low soil compactness and appropriate nitrogen) and T2N1 (High soil compactness and appropriate nitrogen).

Besides modulating aspects of plant growth, soil properties often affect the generation and development of root nodules in legume species. In the present experiment, the number and structure of peanut nodules in soil differing in compactness and nitrogen content were significantly different (Figure 2). Under the condition of an adequate soil N supply, a small number of root nodules formed. Under the condition of low soil N supply, greater soil compaction reduced the number of root nodules. According to the examined root nodule sections, although the change in soil compaction did not significantly influence the size of root nodules, it had a pronounced effect on the number of bacteria contained in each. The central bacterial-containing cell zone of the root nodule was significantly smaller in compacted soil than in uncompacted soil.

### Transcriptome profiling of peanut root under low N and soil compaction stresses

**Figure 3:**
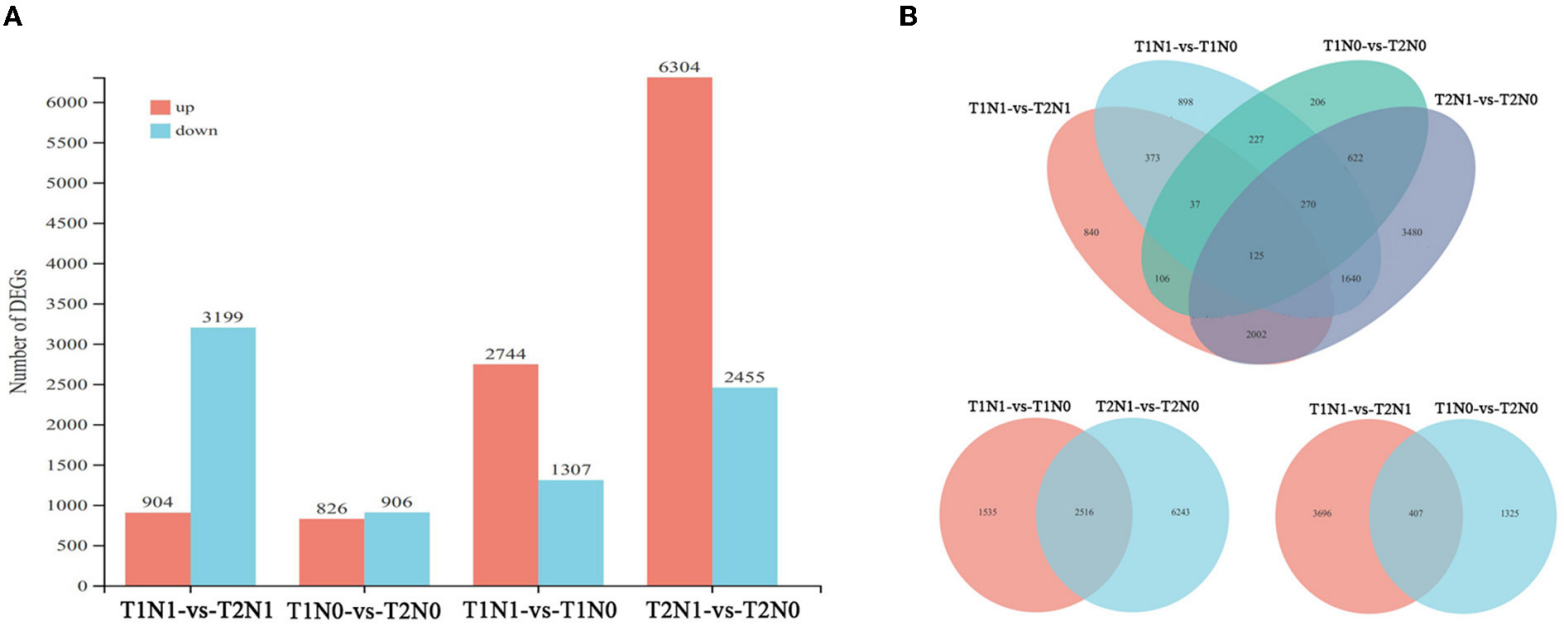
Distributions of differentially expressed genes (DEGs) in all comparisons. **(A)** The number of up-regulated and down-regulated DEGs in each comparison. Red indicates up-regulated genes and blue indicates down-regulated genes. **(B)** Venn diagram for those DEGs in the comparisons related to N deficiency (T1N1-vs-T1N0 and T2N1-vs-T2N0) and soil compaction (T1N0-vs-T2N0 and T1N1-vs-T2N1). T1N0 (Low soil compactness and nitrogen deficiency), T2N0 (High soil compactness and nitrogen deficiency), T1N1 (Low soil compactness and appropriate nitrogen) and T2N1 (High soil compactness and appropriate nitrogen).

We identified a total of 75,810 genes, of which 18,645 were differentially expressed in comparisons of the four treatment combinations (Figure 3A). Except for 39 genes, the vast majority of differential genes were specifically expressed in different comparisons. As seen in Figure 3B, among all comparisons, the highest number of DEGs was found in T2N1-vs-T2N0—nearly double those in T1N1-vs-T1N0. This implied the superimposition of soil compaction stress had a significant effect on the growth and development of peanut root when compared with N deficiency stress alone, while superimposing two stress factors caused starker changes in the expression of peanut root genes. Through a comparative analysis of DEGs, it was revealed that 1535 and 6243 genes uniquely expressed under different N levels of the same soil compaction level (respectively, T1N1-vs-T1N0 and T2N1-vs-T2N0), and another 2516 genes were co-expressed in these two comparisons. These 2516 genes might be chiefly related to N metabolism and designated here as N deficiency-response genes. Likewise, for DEGs under different levels of soil compaction—T1N1-vs-T2N1 and T1N0-vs-T2N0—for the same soil N content, respectively 3696 and 1325 genes were expressed, with another 407 genes co-expressed in these two comparisons. These 407 genes were thus defined as soil compaction-response genes. To verify the validity of the transcriptome data, 12 genes were randomly selected and subjected to qRT-PCR. The Pearson correlation coefficient was used to calculate the correlation coefficient between the TPM value of RNA-seq and qRT-PCR value. The results indicate a strong correlation between these two approaches. Their resulting expression patterns were basically consistent with the transcriptome sequencing data (Supplementary Figure S2), confirming the reliability of the latter used in this study.

### DEGs' enrichment analyses

**Figure 4:**
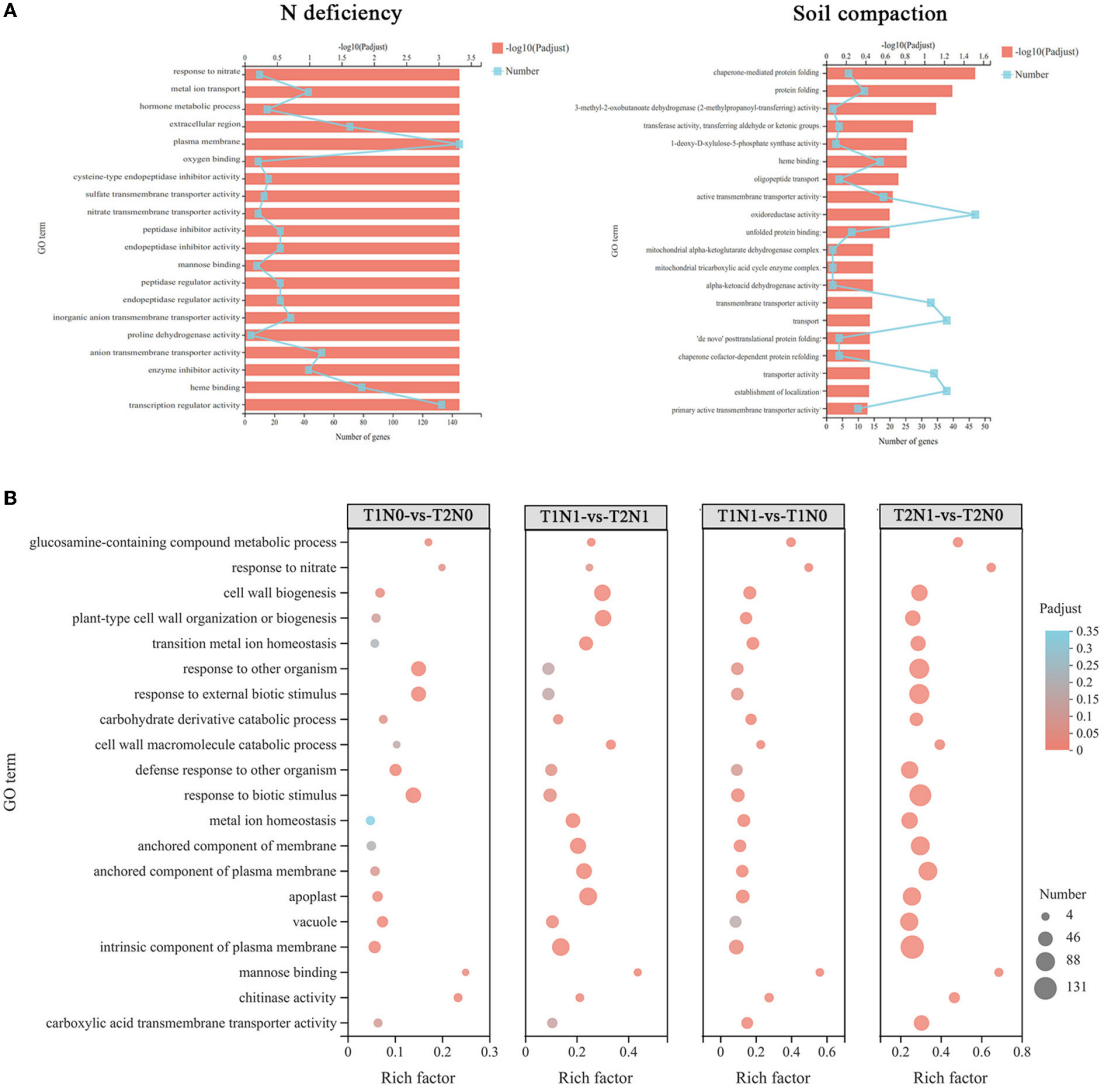
GO enrichment analysis of DEGs (differentially expressed genes) in different sets. The color key represents the corrected *p*-value of enriched GO terms. A high DEGs-enrichment degree is shown in red, and a low DEGs-enrichment degree is shown in blue. The size of the points is proportional to the number of DEGs. **(A)** GO enrichment analysis of those DEGs related to soil compaction stress and N deficiency stress. **(B)** GO enrichment analysis of DEGs related in the four comparisons (T1N0-vs-T2N0, T1N1-vs-T2N1, T1N1-vs-T1N0 and T2N1-vs-T2N0). T1N0 (Low soil compactness and nitrogen deficiency), T2N0 (High soil compactness and nitrogen deficiency), T1N1 (Low soil compactness and appropriate nitrogen) and T2N1 (High soil compactness and appropriate nitrogen).

Next, to explore their function, the N deficiency-response genes were subjected to GO enrichment analysis. As shown in Figure 4A, we found that “response to nitrate” was the most enriched GO term, and the “plasma membrane” was also significantly enriched. Analyzing the soil compaction-response genes revealed they were mainly related to the oxidative stress response and energy metabolism-related GO terms, such as “oxidoreductase activity”, “3-methyl-2-oxobutanoate dehydrogenase (2-methylpropanoyl-transferring) activity”, “mitochondrial metabolism”, and the “mitochondrial alpha-ketoglutarate dehydrogenase complex”.

The GO functions of DEGs in the four different comparisons were analyzed (Figure 4B). Evidently, under different soil compaction at the same soil N content (i.e., T1N1-vs-T2N1, T1N0-vs-T2N0), the functions of DEGs in these two comparisons were mainly enriched in “oxidoreductase activity”, “external stimulus response”, and “transmembrane transporter activity” pathways. Under the same level of soil compaction but differing N stress conditions (i.e., T1N1-vs-T1N0, T2N1-vs-T2N0), the DEGs were mainly enriched in GO terms related to carbohydrate and protein metabolism, such as “carbohydrate derivatives catabolism” and “amino acid transport”. It is worth noting that the higher the soil compactness under N deficiency conditions, the greater the enrichment of DEGs in most GO terms, indicating that the dual stress of soil compaction and N deficiency caused greater effects on peanut roots than the single N deficiency factor stress.

**Figure 5:**
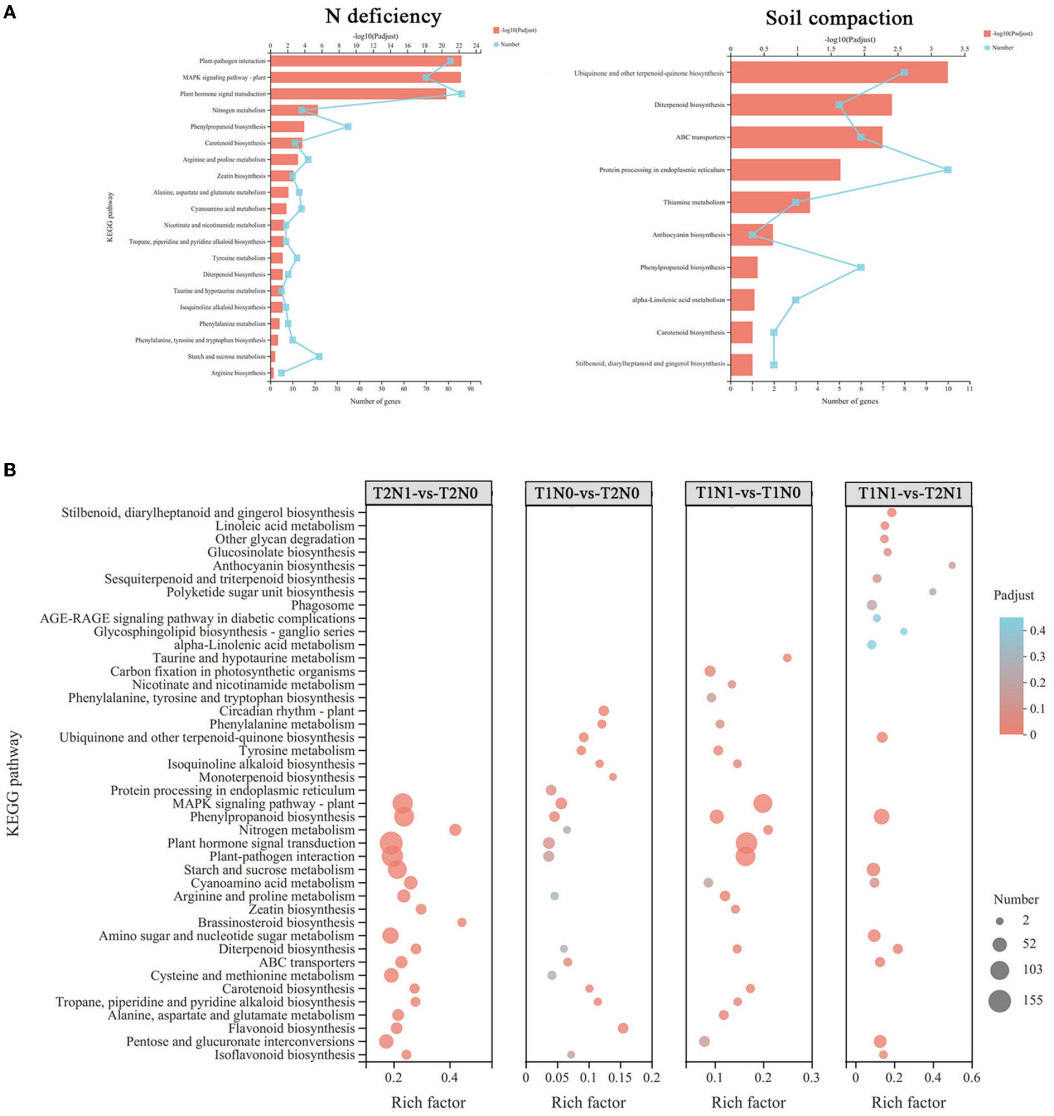
KEGG enrichment analysis of DEGs in different sets. The color key represents the corrected *p*-value of enriched KEGG pathways. The high DEGs-enrichment degree is shown in red and low DEGs-enrichment degree is shown in blue. The size of the points is proportional to the number of DEGs. **(A)** KEGG enrichment analysis of those DEGs related to soil compaction stress and N deficiency stress. **(B)** KEGG enrichment analysis of DEGs in the four comparisons: T1N0-vs-T2N0, T1N1-vs-T2N1, T1N1-vs-T1N0, and T2N1-vs-T2N0. T1N0 (Low soil compactness and nitrogen deficiency), T2N0 (High soil compactness and nitrogen deficiency), T1N1 (Low soil compactness and appropriate nitrogen) and T2N1 (High soil compactness and appropriate nitrogen).

According to the results from the KEGG enrichment analysis of N deficiency-response genes, these were mainly associated with pathways of nitrogen metabolism, amino acid metabolism, and carbohydrate metabolism. Similarly, under different soil N conditions with the same level of soil compaction, the DEGs were found to mainly enriched in the above-mentioned pathways, such as amino acid metabolism (e.g., “arginine and proline metabolism”; “alanine, aspartate, and glutamate metabolism”) and carbohydrate metabolism (e.g., “glycolysis/gluconeogenesis”; “pentose and glucuronate interconversions”). More DEGs were enriched in these metabolic pathways in the T2N1-vs-T2N0 than T1N1-vs-T1N0 comparison, indicating that the superimposition of soil compaction stress enhanced amino acid metabolism and carbohydrate metabolism in peanut roots compared with single N deficiency stress. The KEGG enrichment results for soil compaction-response genes indicated they were mainly enriched in the metabolism pathways of antioxidant secondary metabolites (e.g., “anthocyanin biosynthesis”, “phenylpropanoid biosynthesis”) and lipid metabolism. The number of DEGs enriched in these regulatory pathways was higher in the T1N0-vs-T2N0 than in the T1N1-vs-T2N1 comparison. This implicated antioxidant secondary metabolism and lipid metabolism as important metabolic pathways in peanut roots in their response to soil compaction stress, with N deficiency exacerbating that stress effect. These results showed that amino acid metabolism and sugar metabolism in peanut roots played very important roles in responding to compounded stresses of soil compaction and N deficiency. More genes were involved in peanut root metabolic processes in the coexistence of two stress factors than in the presence of a single N deficiency stress or soil compaction stress (Figure 5).

### Identification of TF genes in response to low N and soil compaction stresses

**Figure 6:**
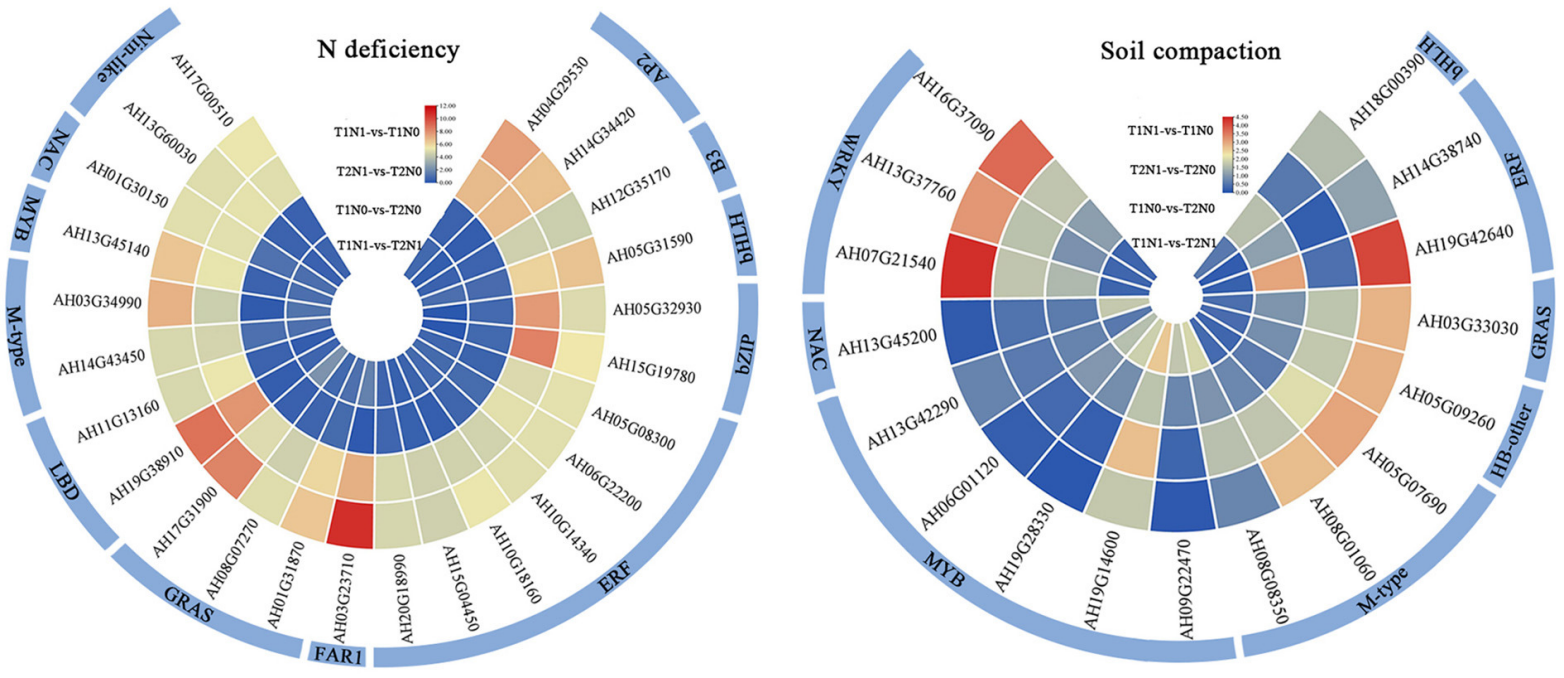
Heatmap of the transcription factor genes response to N deficiency and soil compaction stress in the four comparisons: T1N0-vs-T2N0, T1N1-vs-T2N1, T1N1-vs-T1N0, and T2N1-vs-T2N0. T1N0 (Low soil compactness and nitrogen deficiency), T2N0 (High soil compactness and nitrogen deficiency), T1N1 (Low soil compactness and appropriate nitrogen) and T2N1 (High soil compactness and appropriate nitrogen). The color key corresponds to the calculated log_2_ (fold-change of the TPM value). The N deficiency response-related TF genes that exhibited more than a 20-fold difference in the comparisons T1N1-vs-T1N0 and T2N1-vs-T2N0 are plotted.

Transcription factors (TFs) are important regulators in plant growth and development. In this study, a total of 3526 TF genes belonging to 48 transcription factor families were identified (Supplementary Table S2, Figure 6). Among these, 211 TF genes of 25 TF families were identified in response to N deficiency stress. A significant proportion of TFs (22.2%) were distributed in the ERF family, suggesting that ERF family transcription factors might play a key role in regulating N deficiency stress in peanut roots. Notably, we also identified five members of the Nin-like family of TFs closely related to the symbiotic nodules in this collection, of which three Nin-like family members (AH08G02220, AH13G60030, and AH17G22700) were more prevalent in T2N1-vs-T2N0 than T1N1-vs-T1N0. The down-regulated expression of those three TF genes might mean they are crucial regulators, capable of modulating the impaired root nodule development in peanut plants grown in high-level compacted soil. Additionally, 17 TFs of 9 TF families were identified in response to soil compaction stress. One ERF transcription factor gene (AH19G42640) was found responsive to both soil compaction and N deficiency stresses; hence, this gene might be a key TF regulating the resistance of peanut roots to the compounded stresses of soil compaction and N deficiency.

### Metabolome analysis of peanut roots under low N and soil compaction stresses

**Figure 7:**
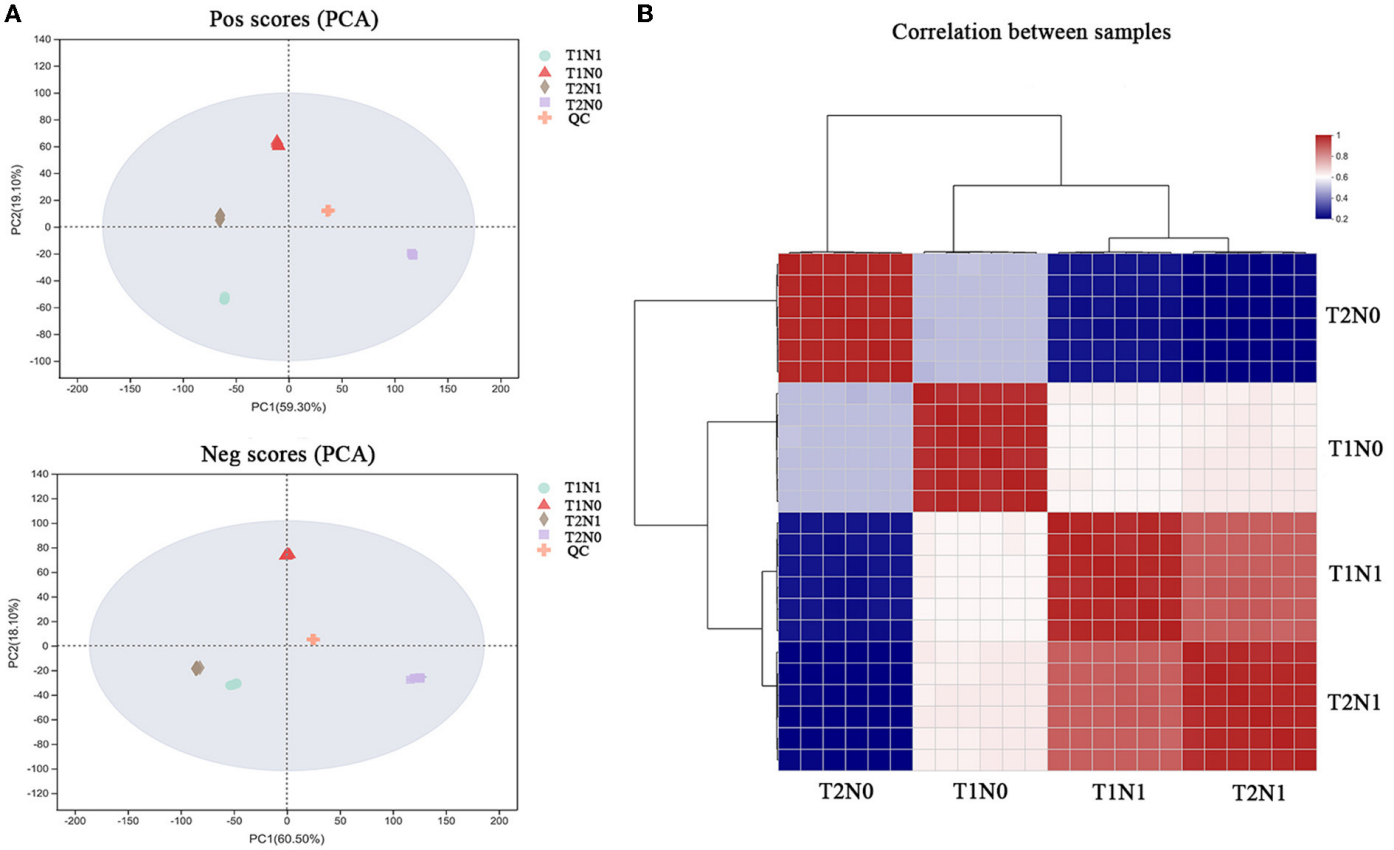
Metabolic profiling results of the peanut root under four experimental conditions (treatment combinations: T1N0, T2N0, T1N1, T2N1). T1N0 (Low soil compactness and nitrogen deficiency), T2N0 (High soil compactness and nitrogen deficiency), T1N1 (Low soil compactness and appropriate nitrogen) and T2N1 (High soil compactness and appropriate nitrogen). **(A)** PCA plot. **(B)** Hierarchical clustering analysis for the metabolites based on their abundances.

**Figure 8:**
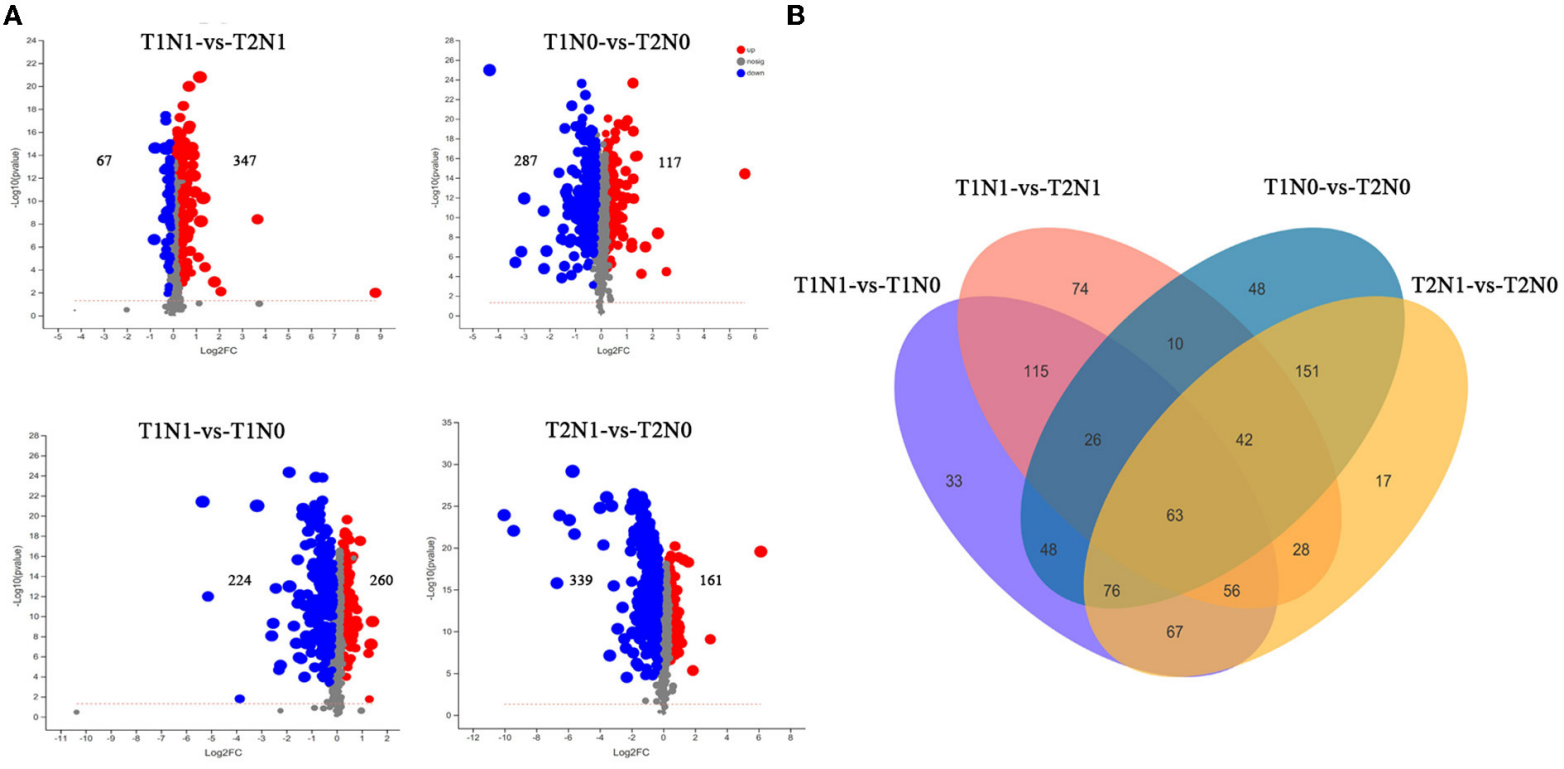
Differentially accumulated metabolites (DAMs) in the four comparisons: (T1N0-vs-T2N0, T1N1-vs-T2N1, T1N1-vs-T1N0, and T2N1-vs-T2N0). **(A)** Number of DAMs in four different comparisons. **(B)** Venn diagram for DAMs in four different comparisons. T1N0 (Low soil compactness and nitrogen deficiency), T2N0 (High soil compactness and nitrogen deficiency), T1N1 (Low soil compactness and appropriate nitrogen) and T2N1 (High soil compactness and appropriate nitrogen).

Metabolites are direct participants in plant physiological and biochemical reactions, whose change in the metabolome under stressful conditions can largely reflect the response and defense of a plant to that adversity. The principal component analysis (PCA) was applied to the metabolomic sequencing data of peanut roots, which showed that different samples under the same stress condition were clustered together, whereas the samples under different treatment conditions were dispersed (Figure 7), indicating that the soil compaction and nitrogen content significantly affect root metabolism in peanut. As Figure 8 shows, by clustering the expression of peanut root tissue samples under different treatment conditions, we found that N deficiency stress had a greater effect than soil compaction stress upon the accumulation of metabolites in peanut roots. These metabolites were simultaneously significantly altered under the dual conditions of soil compaction and N deficiency stresses. In this study, a total of 1175 metabolites were identified and 484 (260 up-regulated, 224 down-regulated), 464 (177 up-regulated, 287 down-regulated), 500 (161 up-regulated, 339 down-regulated) and 414 (347 up-regulated, 67 down-regulated) metabolites exhibited differential accumulation in the T1N1-vs-T1N0, T1N0-vs-T2N0, T2N1-vs-T2N0, and T1N1-vs-T2N1 comparisons, respectively, according to a certified VIP score (VIP > 1) and *p*-value < 0.05 criteria (Figure 8A). As Figure 8B shows, 64 metabolites differentially accumulated in all four comparisons, implying that the metabolism of peanut roots in response to soil compaction and N deficiency stresses comprised joint processes involving multiple substances. In addition, 33, 74, 48, and 17 metabolites accumulated specifically in comparisons of T1N1-vs-T1N0, T1N1-vs-T2N1, T1N0-vs-T2N0, and T2N1-vs-T2N0, respectively. Overall, 262 metabolites accumulated in both comparison of T1N1-vs-T1N0 and T2N1-vs-T2N0 comparisons, and these metabolites may be closely related to N deficiency stress of peanut roots, thus defined here as N deficiency stress-related metabolites. Conversely, 141 metabolites accumulated in both comparisons of T1N1-vs-T2N1 and T1N0-vs-T2N0, these are defined here as soil compaction stress-related metabolites.

### DAMs' related metabolic pathway analysis

**Figure 9:**
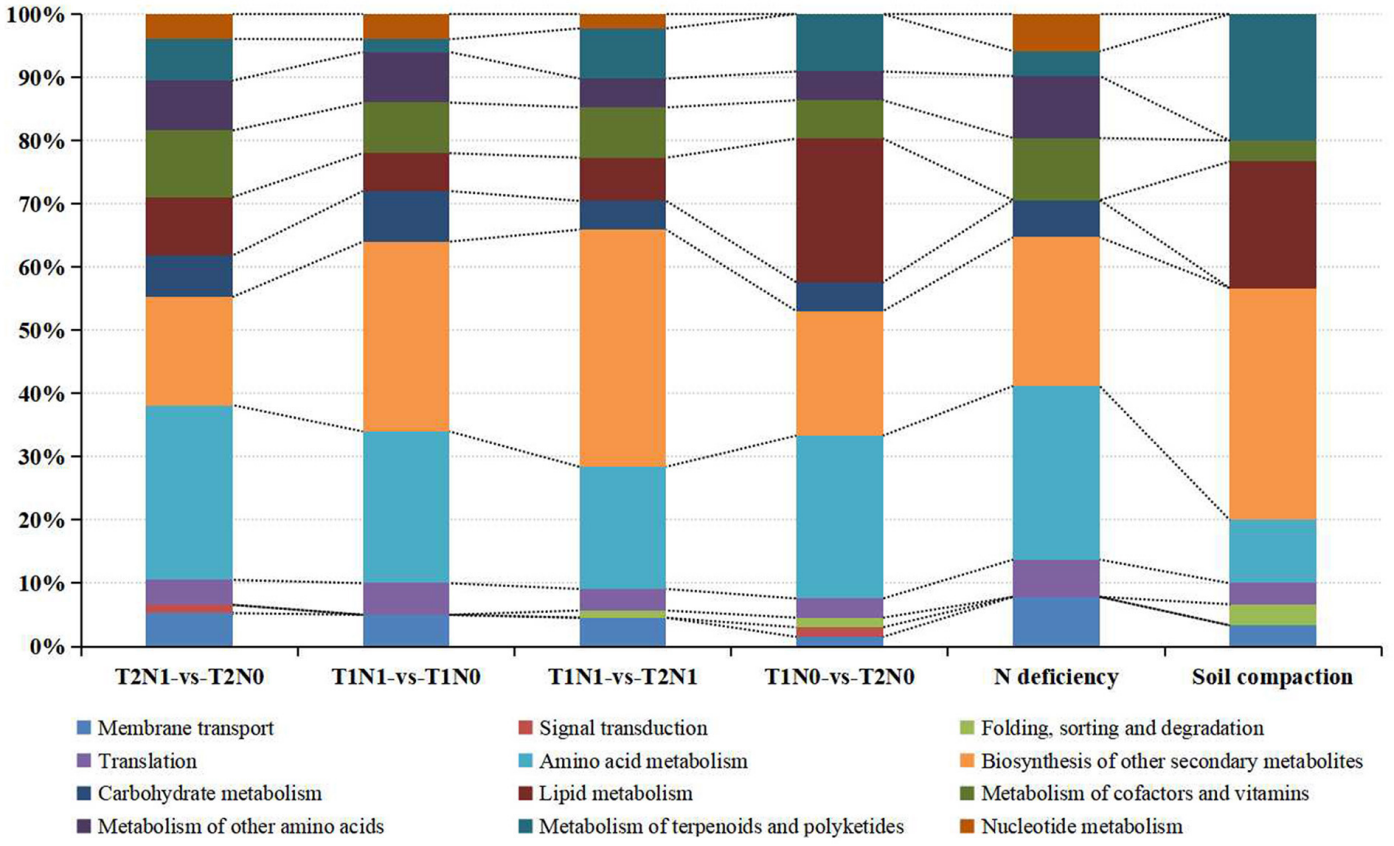
KEGG classifications of DAMs in different sets.

Metabolomics is widely used in plant science to provide a more intuitive understanding of the mechanisms by which plants respond to the magnitude and type of stress, in that minuscule changes in gene and protein expression are amplified at the metabolic scale. As Figure 9 shows, compared with T1N1-vs-T1N0, more known differential metabolites were accumulated in comparison to T2N1-vs-T2N0, especially the metabolites associated with lipid metabolism. This suggested that the superimposition of soil compaction stress caused peanut roots under N deficiency stress to accumulate more metabolites in response to the soil compaction stress. Accumulation of lipid metabolites under N deficiency conditions is an important phenomenon in peanut roots in response to soil compacting stress. KEGG analysis of N deficiency stress-related metabolites for all comparisons and soil compaction stress-related metabolites showed that, compared to other comparisons, under N deficiency stress conditions (T1N1-vs-T1N0, T2N1-vs-T2N0 for N deficiency stress-related metabolites), a significant proportion of metabolites were assigned to amino acid metabolism and carbohydrate metabolism pathways. Under the soil compaction stress (T1N1-vs-T2N1, T1N0-vs-T2N0 for soil compaction stress-related metabolites), a considerable amount of metabolites were assigned to the biosynthesis of other secondary metabolites and lipid metabolism pathways. It is noteworthy that under soil compaction stress, differential metabolites in peanut roots were significantly enriched in isoflavonoid biosynthesis and flavonoid biosynthesis according to the KEGG enrichment, because this suggested that flavonoids and flavonoid metabolites might play an important role in response to soil compaction stress (Supplementary Table S3).

### Integrated analysis of the transcriptome and metabolome data

**Figure 10:**
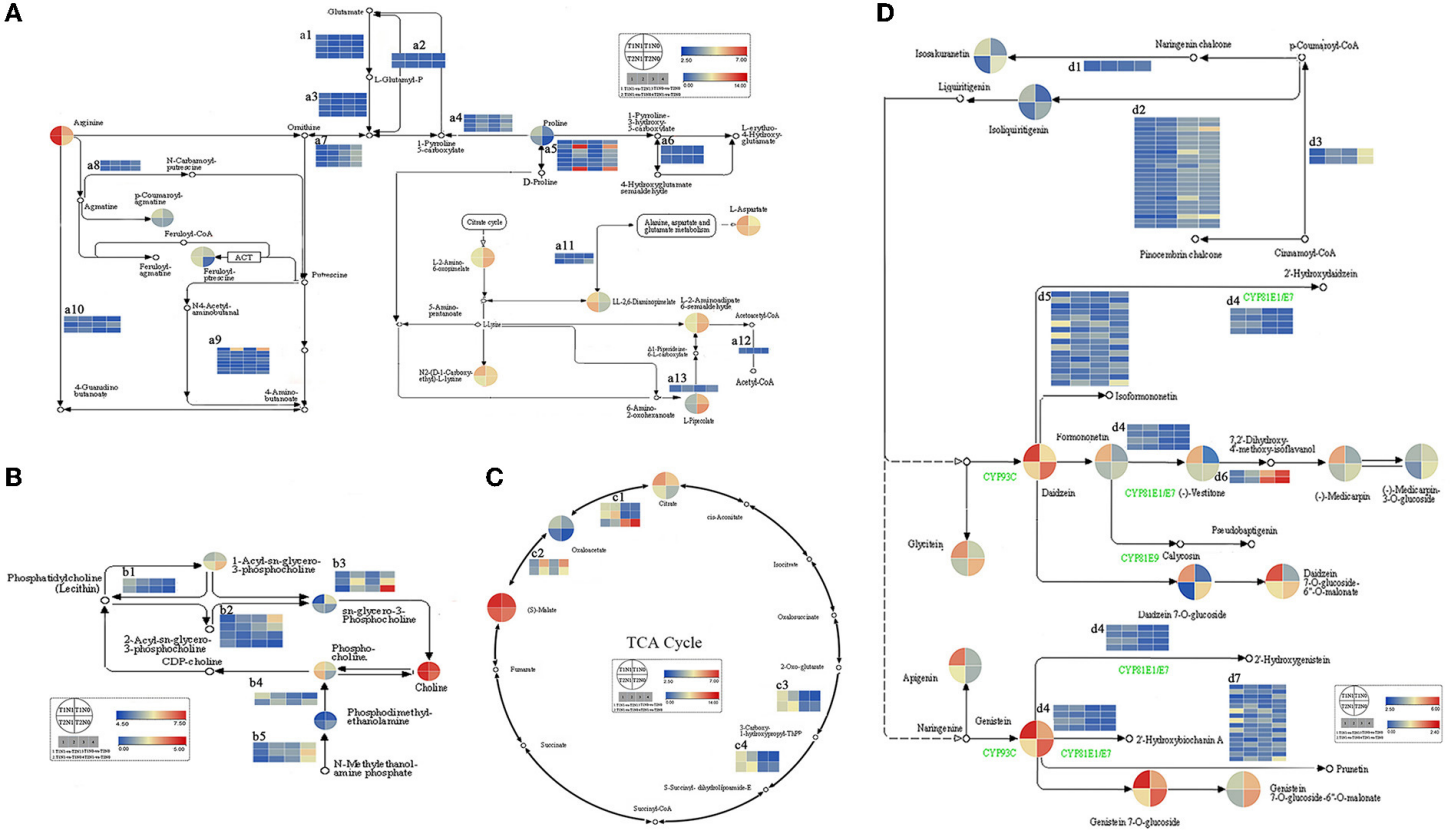
The main metabolic pathways related to soil compaction and N deficiency stress. **(A)** Amino acid metabolism pathway. **(B)** Lipid metabolism. **(C)** TCA cycle. **(D)** Flavonoid and isoflavone metabolic pathway. The color corresponds to the relative abundance of DAMs and fold-change of DEGs in the four comparisons of these pathways. Lower case letters indicate the set of genes involved in this regulatory pathway, as detailed in Supplementary Table S4.

Given that many DEGs and DAMs in the same comparison were assigned to the same metabolism pathway, their correlations were analyzed using the KEGG database to better understand their regulatory network (Figure 10, Supplementary Table S4). Based on the comparative transcriptome analysis result, many DEGs responded to N deficiency (genes in comparison T1N1-vs-T1N0 and T2N1-vs-T2N0) with significantly enriched amino acid metabolism and energy metabolism pathways. The number of genes involved in these two pathways tended to increase as soil compactness increased. Similar to transcriptome analysis, under N deficiency, metabolites associated with these two pathways exhibited significant differences in accumulation. The GO and KEGG enrichment analyses showed that many differential metabolites and differential genes in peanut roots were associated with amino acid metabolism and the TCA cycle under the N deficiency condition. A well-functioning metabolic process of amino acids is fundamental to normal plant growth. The abnormal accumulations of proline and lysine that occurred under N deficiency stress were likely due to the significant differential expression of 39 genes in the amino acid pathway. The TCA cycle is an important pathway by which plants generate energy. In the present study, seven genes related to the TCA cycle exhibited significant differential expression levels, subsequently resulting in the diminished accumulation of key metabolites (citrate, oxaloacetate, and malate) of the TCA cycle.

Under soil compaction stress, KEGG enrichment analysis revealed that DEGs in T1N1-vs-T1N0 and T2N1-vs-T2N0 were significantly enriched in pathways of lipid metabolism and secondary metabolite synthesis (especially the flavonoid biosynthesis pathway and isoflavonoid biosynthesis). In the results of metabolomic analysis, a fair amount of metabolites were assigned to the biosynthesis of other secondary metabolites (especially flavone and isoflavone) and lipid metabolism pathways were abnormally accumulated. Accordingly, the significantly divergent expressions of eight genes related to lipid metabolism and thirty-eight genes linked to flavone and isoflavone metabolic pathways might have contributed to the changes in the accumulation of lipid, flavone, and isoflavone in peanut root under soil compaction stress.

## Discussion

Peanut is an important oil crop worldwide, and recent studies have shown that soil compaction stress and N deficiency have become important factors limiting the growth and development of peanut crops (Shen et al., 2016). The root system is the most direct organ capable of nutrient uptake and perceiving soil compaction stress, being closely related to the shoot growth and yield formation of plant crops (Wang et al., 2019). The levels of soil compaction and nutrient status generally alter the normal growth of peanut plants by directly influencing the development of their root system. Therefore, it is particularly important to investigate the mechanism by which the peanut root system responds to soil compaction and N deficiency in tandem. In this study, we analyzed the changes in differential genes, pertinent metabolic pathways, and differential metabolites in peanut root systems under different levels of soil compaction and N deficiency stresses, by integrating transcriptomics and metabolomics.

Metabolites are direct participants in the physiological and biochemical reactions of plants, and changes in metabolites convey how they are responding to one or more environmental stress factors (Botta et al., 2010; Xin et al., 2019). Primary metabolites are central to plant physiological metabolism and fulfill very critical roles in plant growth and development processes, in addition to stress resistance. Research has shown that primary metabolites, such as amino acids, sugars, and lipids are significantly altered in plants contending with adverse conditions (Bolton, 2009). However, no equivalent study has yet to characterize the primary metabolite changes in peanut root tissues under soil compaction and N deficiency stresses. Amino acids are the units of protein synthesis and the precursors of N metabolism in cells. Amino acid metabolism is closely linked with abiotic stress responses of plants in many species (Verslues and Juenger, 2011; Lei et al., 2015; Chen et al., 2021). In the present study, we found that a significant proportion of N deficiency- and soil-compaction-responsive metabolites were assigned to the amino acid metabolism pathway. Lysine, proline, and arginine were the major DAMs responding to these two abiotic stresses in the different treatment comparisons. Recent research found that abiotic stress could increase the amount of free lysine, whose metabolism was involved in the plant stress response chiefly via saccharopine pathways (Arruda and Barreto, 2020). In maize, lysine could inhibit proline and pipecolate production by inducing the saccharopine pathway under conditions of salt stress (Kiyota et al., 2015). In our study, the lysine in peanut roots showed a significant accumulation effect under the single factor of N deficiency stress or soil compaction stress. The accumulation of lysine further increased when these two stresses were compounded. Lysine metabolism might be a paramount pathway in peanut roots' response to N deficiency and soil compaction stresses. In addition, proline accumulation was reduced under N deficiency and soil compaction stresses, perhaps because of lysine accumulating in peanut roots under stressful conditions, which would have induced the saccharopine pathway and thus catabolized proline. By analyzing the transcriptome data, numerous genes related to the amino acid metabolism process exhibited significant differential expressions under stress. For example, under N deficiency, 29.7% of proline and arginine and 25% of lysine metabolism pathway-related genes were significantly up-regulated in peanut roots in uncompacted soil, with up to 63.8 and 56.2% of genes involved in those metabolic pathways also significantly up-regulated in compacted soil. The transcriptome results were consistent with the metabolome data. In general, the amino acid metabolic pathway is the key underpinning response of peanut roots to N deficiency and soil compaction stresses. To resist the stress damage caused by soil compaction stress to peanut root growth, it may rely on augmenting amino acid metabolism. The exact mechanisms of lysine, proline, and arginine accumulations in relation to peanut's ability to resist damage caused by N deficiency and soil compaction stresses warrants more investigation.

The carbohydrate pathway not only provides ample energy for plants' development, but also serves as a crucial regulatory pathway enabling them to adapt to adverse stresses (Zhang et al., 2017). As the core pathway of carbohydrate metabolism in plant cells, the tricarboxylic acid (TCA) cycle is fundamental to cellular energy production, operating with the carbohydrate biosynthesis pathway to maintain carbon homeostasis in plants under various stress conditions. Previous studies have proved that the TCA cycle in plant roots is repressed by N starvation (Xin et al., 2017; Li et al., 2021). In our study of peanut, the accumulation of principal metabolites in the TCA pathway—such as citric acid, malic acid, and oxoglutaric acid—exhibited a downward trend under N deficiency conditions, suggesting that N deficiency stress could limit or halt carbohydrate metabolism in peanut roots. Furthermore, a recent study showed that the TCA cycle pathway in roots could be significantly inhibited under high soil compaction (Wang et al., 2019). In the present study, we also found that soil compaction could reduce carbohydrate accumulation in peanut roots. Under an N deficiency condition, when the soil compaction is greater there are fewer metabolites accumulating in the TCA pathway, strongly implying that superimposing soil compaction stress exacerbates the inhibitory effect of N deficiency stress on the TCA pathway in peanut roots. Consistent with the results of metabolomics, soil compaction and N deficiency stress inhibited the expression of a citrate synthase gene (AH11G01560) by analyzing the transcriptome data, which encodes a major rate-limiting enzyme in the TCA cycle, blocking citric acid synthesis and reducing the accumulation of principal metabolites participating in the TCA cycle. Further, in an earlier study, the accumulation of organic acid metabolites produced during the TCA cycle contributed to the production of peanut root nodules and N fixation (Zuo et al., 2004). The reduced organic acid accumulation caused by the TCA cycle's inhibition in peanut roots could be a reason for the impeded peanut root nodule development in compacted soil. Based on these results, we conclude that N deficiency and soil compaction stresses decrease the expression of major rate-limiting enzyme citrate synthase in the TCA pathway, leading to the reduced accumulation of carbohydrate metabolites, which inhibits the TCA cycle in peanut roots and subsequently affects the energy supply for peanut.

Besides amino acids and sugars, lipids are also important primary metabolites in plants functioning importantly in energy conversion, signal transduction, and stress response processes (Suh et al., 2015). Active changes to membrane lipids in plant roots have been observed under many types of abiotic stress conditions, including temperature stress (Zhao et al., 2021), salt stress (Xu et al., 2021), and nutritional stress (Nakamura, 2013). In legumes, lipid metabolism in the root is crucial for nodule formation and development (Zhang et al., 2020). One of the primary processes by which plants phenotypically adjust to abiotic stresses is by regulating their biomembrane fluidity, a mechanism influenced by both species identity and the components of lipids on the membrane (Li et al., 2016). In peanuts, its phospholipids are composed of phosphatidyl choline (PC), phosphatidyl ethanolamine (PE), phosphatidic acid (PA), phosphatidyl inositol (PI), and lysophosphatidylcholine (LPC), among others (Zhang et al., 2019). Recently, it was reported that soil compaction stress could worsen soil aeration, resulting in an oxygen content reduction in the root region of the plant, so that the accumulation of ROS there interfered with the stability of the biomembrane (Drew et al., 2000). In the present study, we found that the accumulation of a variety of metabolites related to lipid metabolism changes dramatically under soil compaction stress. The differential metabolites and their respective content were further analyzed; most of the main components of membrane lipids, such as phosphatidylcholine, glycerylphosphorylethanolamine, glycerophosphocholine, and LPC accumulated under soil compaction and N deficiency conditions. Phospholipases C (PLC) and phospholipases D (PLD) are the major enzymes involved in the degradation of main components of membrane lipids PC and PE (Xue et al., 2007). In our study, the down-regulation of most PC and PE degradation-related genes (PLD, PLC, and PLA) under soil compaction stress could have contributed to the accumulation in peanut roots of its main component membrane lipids. Previous studies have proved that maintaining cell membrane stability by accumulating the PC and PE content of the membrane is an effective means for plants to resist cell membrane damage (Perlikowski et al., 2016; Wang et al., 2022). We speculated that the greater accumulation of PC and PE might be one of the main strategies of peanut roots when responding to soil compaction and N deficiency stresses.

In recent years, researchers have found that plants can accumulate specific secondary metabolites via some specific mechanisms to resist biological or abiotic stresses. Secondary metabolites play an important role in improving the protection and survival competitiveness of plants and coordinating their relationship with the local environment. Compared with primary metabolites, the generation and changes in secondary metabolites are even more strongly correlated with the environment than primary metabolites (Makkar et al., 2007; Tiwari and Rana, 2015). Studying the relationships between peanut roots' secondary metabolites and soil compaction and N deficiency stresses would provide a novel way to comprehensively understand the linkages between plant physiology and abiotic stress. Flavonoids are phenylpropanoid metabolites that function critically in environmental stress resistance, especially in legumes (Treutter, 2006; Stewart et al., 2010; Xu et al., 2015). Flavonoids in legume root exudates can activate nod gene expression of rhizobia and induce the signal molecule Nod factor, after which the complex flavonoids-Nod protein enables the mutual recognition of root and rhizobium from leguminous crops to promote the nodule formation (Subramanian et al., 2007). Accordingly, obstructing the flavonoid accumulation in roots would result in substantially fewer nodules. For instance, in *Medicago truncatula*, silencing the expression of *CHS* (chalcone synthase) led to its flavonoid deficiency, which then impaired its root nodule formation (Wasson et al., 2006). Soil compaction is long recognized as a common barrier to crop growth in farmland. Studies have shown that both nodule number and nodule nitrogenase activity of legume crops in compacted soil were severely impacted (Siczek and Lipiec, 2011; St-Martin and Bommarco, 2016). Siczek et al. (2013) found that the distribution of nodule area, nodule number, nodule weight, and nodule activity of peas growing in compact soil decreased compared with those in non-compacted soil; however, nodule number, nodule weight, and nitrogenase activity of pea roots all increased when the nod factor (lipo-chitooligosaccharides) was added to the compacted soil. Given the close relationship between the occurrence and development of nodules and flavonoids-Nod protein, we hypothesized that compacted soil exerted strong effects on the occurrence and development of root nodules by affecting the production and metabolism of flavonoids in legumes. In the present study, by classifying the DAMs of peanut in different comparisons, we found that most of the soil compaction stress-related differential metabolites were assigned to the term “biosynthesis of other secondary metabolites'. The vast majority of these were flavonoids and isoflavones. KEGG enrichment analysis of metabolites confirmed that flavonoid and isoflavone metabolites were significantly enriched under soil compaction stress, while almost all flavonoid and isoflavone differential metabolites' accumulation were significantly decreased under soil compaction stress; this suggests the metabolic pathways of flavonoids and isoflavones in peanut roots are significantly affected by soil compaction stress. Analysis of transcriptome data revealed that most of the key enzyme genes (including chalcone synthase, flavonol synthase, and flavonoid 3'-monooxygenase) were down-regulated in flavonoid and isoflavone synthesis pathways in peanut roots under soil compaction stress. This could well explain why the flavonoid metabolites were reduced by soil compaction stress. This reduction of flavonoid compounds produced by root metabolism in compacted soil might be a pivotal reason for the decrease in the number of nodules in peanut under soil compaction stress. Given that N is a fundamentally important nutrient for the growth of leguminous species, researchers have paid much attention to the relationship between the production and accumulation of flavonoid compounds in leguminous plants and the N supply level in soil (Siczek et al., 2013). The isoflavone accumulation in soybean seedling root showed a trend of increasing during the early stage of N deficiency stress (Cho and Harper, 1991). But as the growing period progresses, that isoflavone accumulation in soybean declines to eventually reach a relatively stable level in roots (Sugiyama et al., 2016). In our study, 35 days after the seedling emergence of peanut, its roots under N deficiency stress had accumulated less isoflavone than under the appropriate growth condition. Under the combined stresses of soil compaction and N deficiency, the accumulation of isoflavone featured a slight upward trend, perhaps due to a greater relative N concentration in the root zone microenvironment caused by soil compaction. Based on these results, we hypothesize that flavonoid and isoflavone metabolism are important metabolic pathways in response to N deficiency and soil compaction stress in peanut roots. Flavonoid accumulation in peanut roots decreases in the presence of a single N deficiency stress or soil compaction stress, while the coexistence of both stress factors exhibits the opposite trend in flavonoid and isoflavone accumulation, the mechanism of which needs to be further investigated.

## Conclusion

In summary, the metabolic processes in peanut roots were significantly altered under the stresses of soil compaction and N deficiency. Soil compaction stress mainly affected the metabolic processes of lipid and secondary metabolites, while N deficiency stress mainly affected the amino acid and energy metabolic processes in the peanut roots. Soil compaction stress and N deficiency stress each mainly led to an increase in the content of some amino acids (lysine) and fatty acids (especially the lipid composition of plasma membrane) related to the abiotic stress and inhibited the TCA cycle metabolism in the peanut roots. The synergy of the above two stresses exerted an addictive effect on the alterations in the above metabolic pathways. The accumulation of some secondary specific metabolites (e.g., flavonoid and isoflavone) tended to decrease in the presence of a single stressor, however, increased when the two stresses coexisted. Key genes involved in these metabolic pathways also showed corresponding changes based on transcriptome analysis. In this study, the integrated transcriptome and metabolome analysis provided insight into the metabolic and molecular regulatory processes during peanut roots development under soil compaction stress and N deficiency stress, and identified some key genes, metabolites, and metabolic pathways associated with the response to the stresses of soil compaction and N deficiency. This study is important for exploring the mechanisms of abiotic stress resistance in peanut, and provides a theoretical basis for efficient breeding and yield quality improvement in peanut.

## Data availability statement

The datasets presented in this study can be found in online repositories. The names of the repository/repositories and accession number(s) can be found below: https://www.ncbi.nlm.nih.gov/, PRJNA832039; https://db.cngb.org/, CNP0002915.

## Author contributions

LYa and PS conceived this study and designed the experiment. LYa performed the experiments, analyzed the data, and drafted and revised the manuscript. QW, HL, and LYi participated in performing the experiments and analyzing the data. All authors contributed to the article and approved the submitted version.

## Funding

This work was supported by the National Key Research and Development Program of China (2020YFD1000905), Youth Fund of Shandong Natural Science Foundation (ZR2021QC096), and Talent Project for Agricultural Science and Technology Innovation Engineering of Shandong Academy of Agricultural Sciences (CXGC2021B33).

## Conflict of interest

The authors declare that the research was conducted in the absence of any commercial or financial relationships that could be construed as a potential conflict of interest.

## Publisher's note

All claims expressed in this article are solely those of the authors and do not necessarily represent those of their affiliated organizations, or those of the publisher, the editors and the reviewers. Any product that may be evaluated in this article, or claim that may be made by its manufacturer, is not guaranteed or endorsed by the publisher.

## Supplementary material

Supplementary Table S1 Gene-specific primers used for qRT-PCR.

Supplementary Table S2 Summary of TF genes identified in peanut root. N deficiency response TF genes are marked with blue. Soil compaction response TF genes are marked with yellow. TF gene responsive to those two stresses are marked with red.

Supplementary Table S3 Summary of top ten enriched KEGG pathways in all sets (T1N0-vs-T2N0, T1N1-vs-T2N1, T1N1-vs-T1N0, T2N1-vs-T2N0, soil compaction related metabolites and N deficiency related metabolites). The top 10 enriched pathways are based on the p-value. T1N0 (Low soil compactness and nitrogen deficiency), T2N0 (High soil compactness and nitrogen deficiency), T1N1 (Low soil compactness and appropriate nitrogen) and T2N1 (High soil compactness and appropriate nitrogen).

Supplementary Table S4 The relative abundance of genes and metabolites in main metabolic pathways related to soil compaction and N deficiency stress.

Supplementary Figure S1 Phenotype of whole plant at the time of harvest.

Supplementary Figure S2 Real-time qPCR validation of 12 genes in peanut. Shown are their relative expression levels; bars are the standard deviation. The AhACT11 gene was used as an internal control to normalize the expression data. The Pearson correlation coefficient was used to calculate the correlation coefficient between the TPM value of RNA-seq and qRT-PCR value for each gene and fold change (T2N1/T1N1 or T1N0/T1N1 or T1N0/T2N1 or T2N0/T1N0) value of TPM and normalized qPCR for 12 genes. The results indicate a strong correlation between these two approaches. The correlation coefficient (R2) is indicated in this figure.
