# Supplementary figures and images for: Integrated analyses of transcriptome and metabolome provides new insights into the primary and secondary metabolism in response to nitrogen deficiency and soil compaction stress in peanut roots
Source: Front Plant Sci. 2022 Sep 28;13:948742. doi: 10.3389/fpls.2022.948742 (PMC9554563; doi:10.3389/fpls.2022.948742)

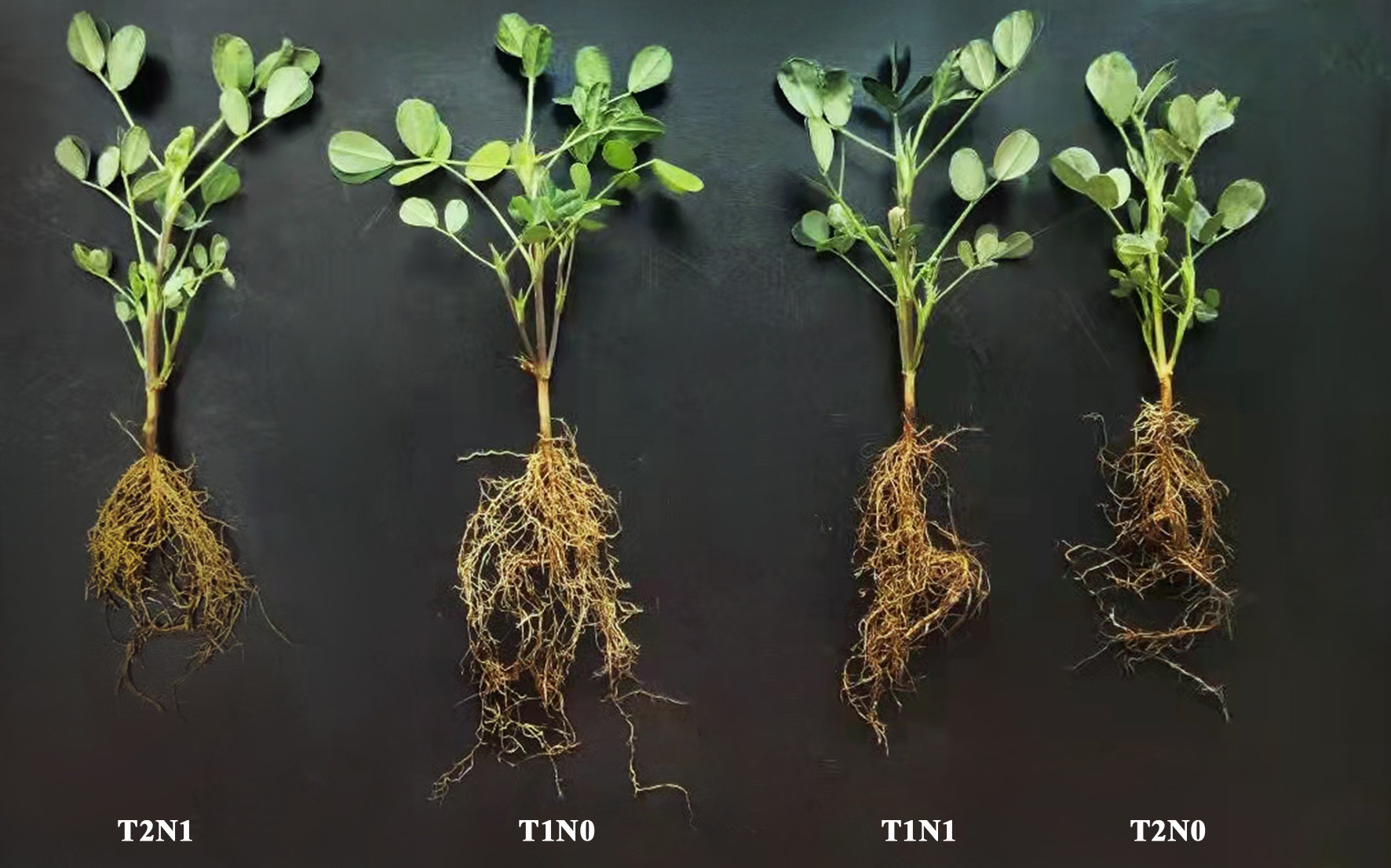

Supplement: Supplementary Figure S1 — Phenotype of whole plant at the time of harvest. [file Image_1.TIF]

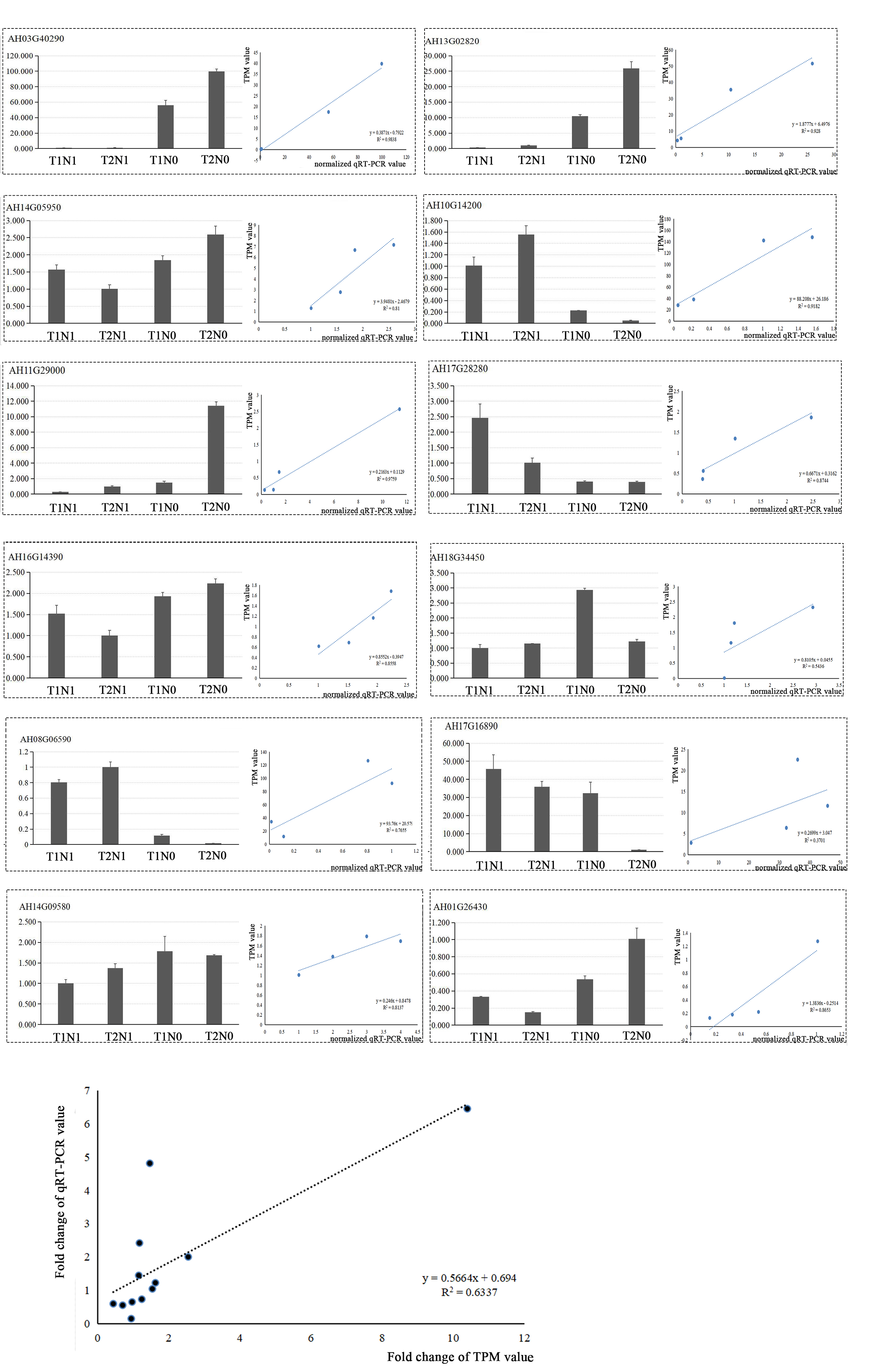

Supplement: Supplementary Figure S2 — Real-time qPCR validation of 12 genes in peanut. Shown are their relative expression levels; bars are the standard deviation. The AhACT11 gene was used as an internal control to normalize the expression data. The Pearson correlation coefficient was used to calculate the correlation coefficient between the TPM value of RNA-seq and qRT-PCR value for each gene and fold change (T2N1/T1N1 or T1N0/T1N1 or T1N0/T2N1 or T2N0/T1N0) value of TPM and normalized qPCR for 12 genes. The results indicate a strong correlation between these two approaches. The correlation coefficient (R2) is indicated in this figure. [file Image_2.TIF]
